# Supplementary material for: Adeno-Associated Virus-like Particles’ Response to pH Changes as Revealed by nES-DMA
Source: Viruses. 2023 Jun 13;15(6):1361. doi: 10.3390/v15061361 (PMC10302287; doi:10.3390/v15061361)
Supplement: Supplementary file 1 [file viruses-15-01361-s001.zip › viruses-2405767-supplementary.pdf]

MDPI Viruses, *to be submitted* (2023)

Supporting Information

Adeno-associated virus-like particles' response to pH changes as revealed by nES-DMA

Samuele Zoratto[1], Thomas Heuser[2], Gernot Friedbacher[1], Robert Pletzenauer[3], Michael Graninger[3], Martina Marchetti-Deschmann[1], Victor U. Weiss[1]

[1] Institute of Chemical Technologies and Analytics, TU Wien, Vienna, Austria

[2] Electron Microscopy Facility, Vienna BioCenter Core Facilities GmbH, Vienna, Austria

[3] Pharmaceutical Sciences, Baxalta Innovations GmbH (part of Takeda), Vienna, Austria

Keywords: nES GEMMA, DMA, VLP, AAV8, cryo-TEM, gene therapy.

Correspondence: Victor Weiss, Institute of Chemical Technologies and Analytics, TU Wien, Getreidemarkt 9/164, A-1060 Vienna, Austria

E-mail: victor.weiss@tuwien.ac.at

Tel: +43 1 58801 151611

Figure S1

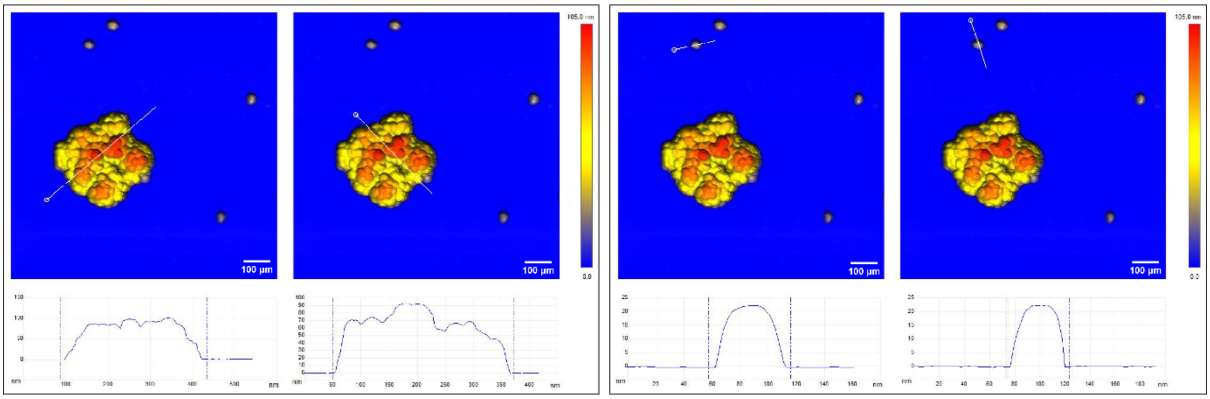

AFM analysis of filled VLPs at pH 5 and relative longitudinal and medial profile section of the cluster (left panel) and one monomeric unit of AAV8 (right panel). Left panel: the profile sections highlight the underlining globular nature of the individual particles forming the cluster.

Figure S2

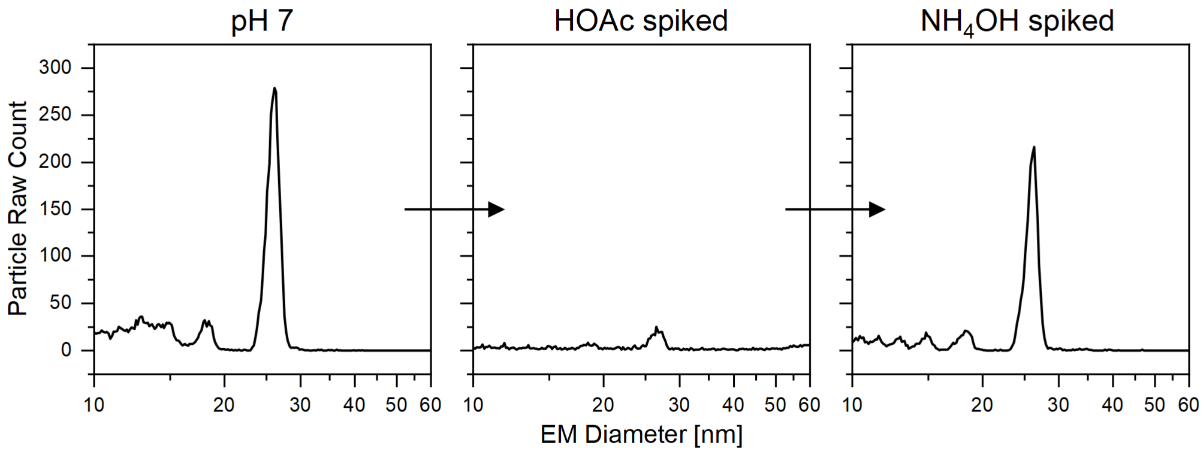

nES GEMMA reversibility experiment of filled VLP preparation. From left to right: in the first panel the VLP preparation has been analyzed at pH 7. In the middle panel, the aliquot was spiked with 1 µL of acetic acid, changing the pH to ~3.6; substantial loss of particle detection is noticeable. In the last panel, 2 µL of ammonium hydroxide are added to the aliquot, changing the pH to ~8.6 and reestablishing the detection of monomeric VLP particles.

Python script:

The following script was employed to extract planar section from the cryo-TEM 2D averaged images.

```
#!/bin/python3

#Python script created by Samuele Zoratto

#contact: samuele.zoratto@tuwien.ac.at

#import of necessary libraries

import numpy as np

import sys

import os

from PIL import Image

import PIL.ImageOps

import csv

import matplotlib.pyplot as plt

from datetime import datetime


#clean the shell

os.system("cls||clear")


#reads filename of the 2D image to convert to 3D and section
```

```
try :
    #check if the file is present from the command line
    image_2D = str(sys.argv[1])
    #check if the filename correspond to an existing file
    if os.path.exists(image_2D):
        print("File: "+image_2D+" found")
    else:
        print(">> Error: File not found! <<\n\nCheck:\n1- Spelling\n2- Absence of blank characters\n3- If the file is in the same directory of this script!\n\nThe program will terminate")
        #terminate the script
        sys.exit(1)
except IndexError :
    #if the file was not provided, it asks for it
    print("No image filename provided in the command line. \nIn the future you can use the following format:\n \n>> python 3D_section_from_2D_image.py [image name] [section number: 0-255] \n \nEnter the filename to use: ", end = "")
    image_2D = input()
    print("\nFilename entered: "+image_2D)
    #check if the filename correspond to an existing file
    if os.path.exists(image_2D):
        print("File "+image_2D+" found! The program will continue\n\n")
```

```
    else:
        print(">> Error: File not found! <<\n\nCheck:\n1- Spelling\n2- Absence of blank characters\n3- If the file is in the same directory of this script!\n\nThe program will terminate")
        #terminate the script
        sys.exit(1)


#reads the section number
try :
    #check if the number has been declared in the command line, if it is valid, and in range
    section_number = str(sys.argv[2])
    if section_number.isnumeric() and -1 < int(section_number) < 256:
        print("Section number in the correct range [0-255]: "+section_number)
    else:
        print(">> Error: Section number not valid! <<\n\nCheck:\n1- Spelling\n2- Number in range [0-255]\n\nThe program will terminate")
        sys.exit(1)
except IndexError :
    #if the section number was not provided, it asks for it
    print("No section number provided in the command line. \nIn the future you can use the following format:\n \n>> python 3D_section_from_2D_image.py [image name] [section number: 0-255] \n \nEnter the section number to use [0-255]: ", end = "")
    try :
```

```
        section_number = input()
        print("\nSection number entered: "+section_number)


        if -1 < int(section_number) < 256 :
            print("Section number accepted")
        else :
            while True :
                print("The section number entered is not valid. Please insert a valid section number [0-255]: ", end = "")
                section_number = input()
                print("\nSection number entered: "+section_number)
                if -1 < int(section_number) < 256 :
                    print("Section number accepted")
                    break
    except ValueError :
        print("\n\n>> ERROR <<\nA non-numeric value has been entered. The program will terminate")
        sys.exit(1)


#generate the filename for the csv file
now = datetime.now()
```

```
datestring = now.strftime("%b-%d-%Y_%H%M%S")
#the filename generated will be in this format: Export_image.jpg_section123_May-30-2022_125500.csv with current date and time
filename = str("Export_" + image_2D + "_section"+section_number+"_"+datestring+".csv")
try :
    file = open(filename,"w",newline="")
    writer = csv.writer(file)
    print("\nCSV file created: "+ filename)
except :
    print("\n\n>> ERROR <<\Impossible to create a file in the directory. Check disk space or writing permissions.\nThe program will terminate")
    sys.exit(1)


#function to write the exported section in the csv file
def printArrayValuesInCSV(tobeprinted):
    for x in range(len(tobeprinted)):
        writer.writerow(tobeprinted[x])


#function to create a list of scattered points from an array
def createScatteredPoints(dataArray):
    dataList = []
```

```
    for x in range(len(dataArray)):
        for y in range(len(dataArray[0])):
            if dataArray[x,y] == True:
                dataList.append([x,y])
    return dataList


#open the 2D image, convert to grayscale, and invert the colors (black = 0 = baseline; white = 255 = highest elevation)
img = PIL.ImageOps.invert(Image.open(image_2D).convert("L")) #L converts to grayscale
#the image is converted in a numpy array, each pixel is converted in a value from 0 to 255
array = np.array(img)
#arrayBool will contain a True value, if the value of the numpy array in position [x,y] equals the section number. In other words, for a given section number (heigth) only those value will be selected.
arrayBool = array == int(section_number)


#conversion of the arrayBool in a list containing the data points (in x and y values) of the selected section
newlist = createScatteredPoints(arrayBool)


#variables for the plot
x,y = zip(*newlist)
```

```
#writing of the array values in the csv file

printArrayValuesInCSV(newlist)

print("Section correctly exported into the csv file")

#close csv file

file.close()



#visualization of the plot

plt.scatter(x,y)

plt.title("Section n°" + str(section_number) + " of "+ str(image_2D))

plt.axis("equal")

print("\nTo terminate the program, close the plot window")

plt.show()
```
